# Draft Genome Sequence of Carbapenem-Resistant Klebsiella pneumoniae XPY20 Collected from a Bloodstream Infection Patient

**DOI:** 10.1128/genomeA.00443-18

**Published:** 2018-05-24

**Authors:** Qiong Chen, Jia-wei Zhou, Sheng-hai Wu, Xiao-hua Meng, Dao-jun Yu, Xian-jun Wang

**Affiliations:** a Department of Laboratory Medicine, Hangzhou First People’s Hospital, Hangzhou, Zhejiang, China; b State Key Laboratory for Diagnosis and Treatment of Infectious Diseases, Collaborate Innovation Center for Diagnosis and Treatment of Infectious Diseases, The First Affiliated Hospital, College of Medicine, Zhejiang University, Hangzhou, China

## Abstract

Bloodstream infections caused by carbapenem-resistant Klebsiella pneumoniae (CRKP) strains have been a severe problem with high clinical costs and high mortality rates. The *bla*_KPC-2_-producing CRKP strain XPY20 was collected from the blood of a patient. The genome characteristics and antimicrobial resistance mechanisms were determined using next-generation sequencing.

## GENOME ANNOUNCEMENT

Klebsiella pneumoniae is a pathogenic Gram-negative bacterium that causes more than 95% of the infections linked to Klebsiella spp. Nowadays, clinical studies have shown that K. pneumoniae is often associated with hospital-acquired infections rather than community-acquired infections and lead to urinary and respiratory tract infections, pneumonia, septicemia, and soft tissue infection (1, 2). Moreover, bloodstream infections caused by CRKP strains often result in high mortality rates (3). Given the abundant utilization of antibiotics, especially the usage of third-generation cephalosporins, carbapenem-resistant K. pneumoniae is a global challenge. In the present study, the carbapenem-resistant strain K. pneumoniae XPY20 was isolated from the blood of an 84-year-old patient with pulmonary infection and anemia. XPY20 was sequenced using a next-generation sequencing system to study its genetic characteristics.

Genomic DNA was extracted using a QIAamp DNA minikit and was then fragmented using Covaris 220. A sequencing library was constructed using an Illumina Nextera library preparation kit following the manufacturer’s instructions. Whole-genome sequencing was performed using the Illumina HiSeq X platform. The removal of low-quality, duplicate, and adapter reads resulted in about 1.5 Gb of clean data, which gave a 279.6-fold average coverage of the genome. The clean reads were assembled *de novo* with Velvet version 1.2.10. Contigs less than 500 bp were trimmed, and the remaining 107 assembled contigs totaled 5.6 Mb with a G+C content of approximately 56.99%.

The genome was annotated using the NCBI Prokaryotic Genome Annotation Pipeline (PGAP) version 4.2 (4), which identified 5,673 coding genes (CDSs) and 95 RNA-coding genes. Multilocus sequence typing (MLST) through the Pasteur Institute’s MLST website (http://bigsdb.pasteur.fr/klebsiella/klebsiella.html) showed that it belonged to sequence type 11. The resistance genes were predicted with ResFinder version 2.1, and the results showed that the strain had resistance genes against classes of antibiotics, including two aminoglycoside resistance genes (*aadA2* and *rmtB*), four beta-lactam resistance genes (*bla*_SHV-11_, *bla*_TEM1B_, *bla*_KPC-2_, and *bla*_CTXM-65_), one fluoroquinolone resistance gene (*qnrS1*), two fosfomycin resistance genes (*fosA* and *fosA3*), one phenicol resistance gene (*catA2*), two sulfonamide resistance genes (*sul1* and *sul2*), one tetracycline resistance gene [*tet*(A)], and one trimethoprim resistance gene (*dfrA14*).

### Accession number(s).

This whole-genome project has been deposited in GenBank under the accession no. PCFS00000000. The version described in this paper is the first version.
